# ShetlandsUAVmetry: unmanned aerial vehicle-based photogrammetric dataset for Antarctic environmental research

**DOI:** 10.1038/s41597-024-03045-1

**Published:** 2024-02-14

**Authors:** Alejandro Román, Gabriel Navarro, Antonio Tovar-Sánchez, Pedro Zarandona, David Roque-Atienza, Luis Barbero

**Affiliations:** 1 Institute of Marine Sciences of Andalusia (ICMAN), Spanish National Research Council (CSIC), Department of Ecology and Coastal Management, 11510 Puerto Real, Spain; 2 University of Cádiz, Department of Earth Sciences, International Campus of Excellence in Marine Science (CEIMAR), 11510 Puerto Real, Spain; 3 King Abdullah University of Science and Technology (KAUST), 23955 Thuwal, Saudi Arabia

**Keywords:** Environmental impact, Geology

## Abstract

The study of the functioning and responses of Antarctica to the current climate change scenario is a priority and a challenge for the scientific community aiming to predict and mitigate impacts at a regional and global scale. Due to the difficulty of obtaining aerial data in such extreme, remote, and difficult-to-reach region of the planet, the development of remote sensing techniques with Unmanned Aerial Vehicles (UAVs) has revolutionized polar research. ShetlandsUAVmetry comprises original datasets collected by UAVs during the Spanish Antarctic Campaign 2021–2022 (January to March 2022), along with the photogrammetric products resulting from their processing. It includes data recorded during twenty-eight distinct UAV flights at various study sites on Deception and Livingston islands (South Shetland Islands, Antarctica) and consists of a total of 15,691 high-resolution optical RGB captures. In addition, this dataset is accompanied by additional associated files that facilitate its use and accessibility. It is publicly accessible and can be downloaded from the figshare data repository.

## Background & Summary

Antarctica, including its continental area and the surrounding Southern Ocean, is one of the most rapidly affected areas by climate change. Therefore, it is imperative and a scientific challenge to comprehend its functioning in order to predict and mitigate risks at both regional and global scales^1,2^. These remote regions of the planet, typically located in rugged and inaccessible sites affected by extreme and changing weather conditions, hinder the deployment of traditional *in-situ* monitoring techniques, which can be dangerous, challenging, and time-consuming^3,4^. The onset of Antarctic remote sensing dates back to 1929 with Hubert Wilkins’ first airplane flight over Deception Island (South Shetland Islands, Antarctica)^5^. Since then, and with the enhancement of satellite remote sensing through technological advancements, there are a wide range of studies available for polar ecosystems^6–10^. However, very high-resolution (VHR) satellite optical data faces some limitations in Maritime Antarctica, such as the almost permanent cloud coverage, its cost, and the fact that VHR imagery is not obtained regularly.

Unmanned Aerial Vehicles (UAVs) have emerged as an intermediate monitoring platform between satellite imagery and ground-based techniques for collecting data in remote and difficult-to-access regions comprising a significant portion of the cryosphere. In such situations, UAVs offer an affordable, flexible, and less intrusive alternative^11–13^, that can be deployed more regularly, overcoming cloud coverage limitations and providing centimetric or even millimetric spatial resolutions^14–16^. Structure-from-Motion (S*f*M) photogrammetry techniques have been successfully applied to UAV datasets to generate final georeferenced orthomosaics and topographic products (including a point cloud, 3D mesh, Digital Terrain Model (DTM), or Digital Surface Model (DSM)) by finding common points between the subsequent overlapped captures through triangulation^17,18^. In Antarctica, research on the use of UAV-based photogrammetric products has focused on several areas, including: (i) elaboration of detailed basemaps^19–21^; (ii) counting fauna individuals and determining their main morphometric features^22–25^; (iii) mapping vegetation^26–28^; (iv) studying glaciers and ice sheets^29–31^; and (v) monitoring landforms and soils^32,33^, many of them are included in Pino & Vieira’s review on the use of UAVs in scientific activities in Antarctica^14^.

**Fig. 1:**
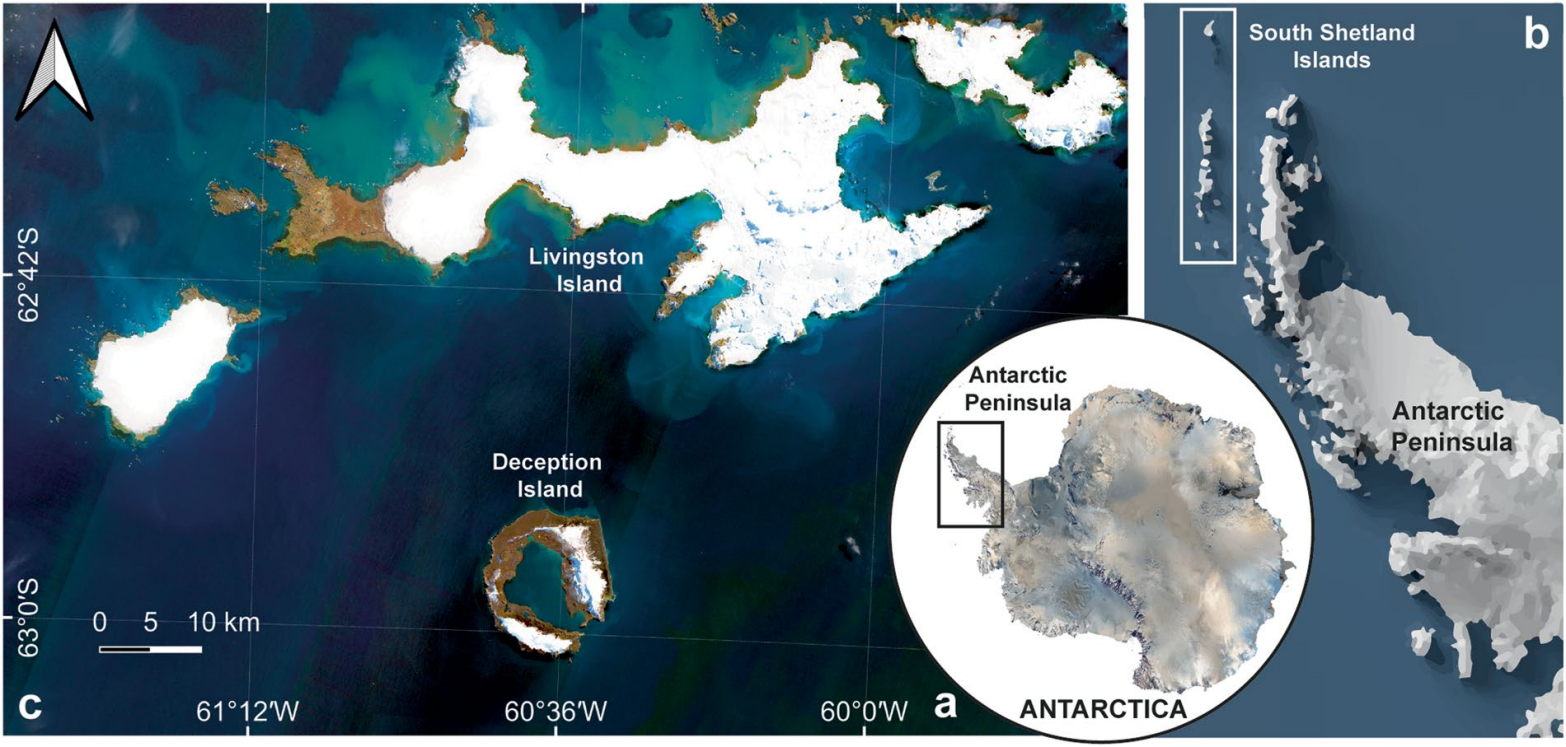
Map showing the locations of: (**a**) the Antarctic Peninsula in a general overview of Antarctica generated with QAntarctica package^74^; (**b**) the South Shetland Islands in the Antarctic Peninsula generated with QAntarctica package^74^; and (**c**) Sentinel 2 A scene of Deception and Livingston Islands in the South Shetland Islands on 17 March 2023.

Here, we present the ShetlandsUAVmetry dataset, comprising the original raw data and the high-resolution photogrammetric products obtained from 28 UAV flights at multiple locations on Deception Island and Livingston Island (South Shetland Islands, Antarctica, Fig. 1) during the Spanish Antarctic Campaign 2021–2022. The Pix4D Mapper software (Pix4D SA, Lausanne, Switzerland, v.4.8.3) was used for processing the UAV captures, employing S*f*M photogrammetry workflow with Real Time Kinematic (RTK) technology for georeferencing accuracy. Considering the difficulties of acquiring data in such a harsh environment and the wide range of scientific applications involving the use of UAV-based photogrammetric products on Antarctica, this dataset offers exceptional quality and serves as a valuable resource for polar research, providing insights into Antarctica’s ecological functioning amidst the current climate change scenario. To the best of our knowledge, the ShetlandsUAVmetry is the first publicly available UAV-based photogrammetric dataset for an Antarctic area. It holds great potential for supporting various research activities, such as (i) the application of artificial intelligence (AI) algorithms for counting wildlife in the main breeding sites; (ii) the extraction of geological information using DSM from a 3D point cloud for the elaboration of high-resolution basemaps or the detailed monitoring of landforms; (iii) the characterization of small and sparse vegetation features, as well the detection of changes in their distribution, in a wide variety of Antarctic ecosystems; (iv) the analysis of coastal erosion rates; and (v) the topographic monitoring of glaciers and ice-sheets to assess melting or subsidence events and mitigate their ecological consequences, among others.

## Methods

### Sites description

Various locations on Deception Island and Livingston Island (South Shetland Islands, Antarctica) were surveyed between January and March 2022 to acquire optical RGB imagery using different sensors aboard UAVs for the application of S*f*M photogrammetry (Tables 1, 2, respectively).

- Deception Island (located between latitudes 62°53′S and 63°01′S, and longitudes 60°29′W and 60°45′W) constitutes the uppermost part of the most active volcano in the South Shetland Islands^34,35^. It is characterized by a central caldera depression with a diameter of 8–10 km, known as Port Foster, which is currently sea-flooded and connected to the open sea through Neptune’s Bellows^35,36^. The primary processes influencing the geomorphology of Deception Island are volcanic activity, resulting in a wide variety of landforms and deposits stemming from small-scale volcanic eruptions recorded in the past two centuries^36–38^, and glacial action, as approximately 57% of the island’s surface is covered by glaciers, partially ice-cored moraines, and areas of glacial ice covered by pyroclasts^39^. Among its multiple structures, Murature formation stands out as a consolidated andesitic lapilli tuff^2^. Due to its scientific significance, the Argentine (Deception) and Spanish (Gabriel de Castilla, BAE GdC) Antarctic research stations are located on the island^40^.
  The inner sector of the caldera concentrates most of the island’s volcanic activity, featuring well-preserved craters, fumarolic emissions, and hot soils in coastal areas, especially in regions between Fumarole Bay and Pendulum Cove, where geothermal activity reaches temperatures exceeding 110 °C^41^. In fact, there is a significant variation in soil temperature between these bays and Whalers Bay, located at the southeastern end of the island, where soil temperatures do not exceed 40–60 °C^42^. In addition, Whalers Bay experienced the influence of anthropic activities, undergoing drastic transformations derived from the presence anchored ships for processing whale oil between 1912 and 1931, which in turn attracted birds to the vicinity due to the presence of whale carcasses^43^. The island’s relief has been also influenced by periglacial activity, with the formation of streams and lakes by melting of ice and snow such as Crater Lake^39^.
  In terms of biodiversity, the island is known for its uncommon plant species, some of which are exceptionally rare, and diverse bryophyte communities associated with geothermal activity^2,44^. In addition, the topographic features of the island make it an ideal location for the establishment of important penguin colonies, such as Vapour Col or Baily Head^16,22^. Vapour Col is characterized by its ice-free surface and its abrupt slope on the southwest coast of the island, and constitutes one of the largest Chinstrap (*Pygoscelis antarcticus*) penguin colonies at Deception Island (population census of 19,177 breeding pairs^45^). Moreover, distinct patches of penguin guano play a significant role in shaping different vegetation communities around these seabird colonies^16^. Baily Head is located in the eastern outer coast and features a largely linear ice cliff with a narrow sandy-gravelly beach at its base^46^. These characteristics have allowed for the settlement of the largest Chinstrap penguin colony on the island (population census of 50,408 breeding pairs^45^), with the green alga *Prasiola crispa* being the most abundant vegetation community in the colony.
- Livingston Island (located between latitudes 62°27′S and 62°48′S, and between longitudes 59°45′W and 61°15′W) is the second-largest of the South Shetland Islands, and the island’s surface predominantly comprises exposed rocks and snow/ice-covered terrain. Byers Peninsula forms the western promontory of the island, featuring the largest area of exposed rock, while the rest of the island consists of an irregular ice cap stretching from Byers Peninsula to McFarlane Strait in the east^47^. However, most of the surveyed areas in this study are concentrated on Hurd Peninsula, located in the southeastern mountainous region of the island, and including some ice-free areas where research stations, such as the Spanish Antarctic station Juan Carlos I (BAE JCI), are established^47,48^.

**Table 1:** Information on UAV surveys, study locations, and flight conditions at study locations on Deception Island.

Location	Key aspects	Central Coordinates	UAV	Sensor	Date	# Images	Flight Time	Weather Conditions	Average GSD (cm/px)	Covered Area (ha)
BAE GdC	Spanish Antarctic Station	62°58′37″S 60°40′31″W	M300	DJI Zenmuse P1	Jan 23th, 2022	730	18 min	Overcast with drizzle	1.80	8.51
Crater Lake	Periglacial lake on a volcanic caldera	62°59′05″S 60°40′05″W	M300	DJI H20T	Jan 24th, 2022	1501	90 min	Overcast	8.30	152.76
Vapour Col	Chinstrap Penguin Colony	62°58′53″S 60°43′39″W	M300	DJI H20T	Jan 26th, 2022	290	34 min	Overcast	6.91	50.69
			M300	DJI L1	Jan 26th, 2022	543	29 min	Overcast	1.43	13.62
Whalers Bay	Thermal Anomalies and Anthropic Impacts	62°59′04″S 60°33′35″W	M300	DJI H20T	Jan 29th, 2022	1665	161 min	Overcast	6.64	270.08
Pendulum Cove	Thermal Anomalies	63°50′45″S 58°22′34″W	M2EA	RGB	Jan 30th, 2022	505	33 min	Overcast and Foggy	0.82	17.44
Murature	Consolidated Andesitic Lapilli Tuff	62°57′56″S 60°42′56″W	M300	DJI H20T	Jan 31st, 2022	358	35 min	Overcast	6.42	81.98
Fumarole Bay	Thermal Anomalies	62°58′21″S 60°42′27″W	M2EA	RGB	Feb 1st, 2022	1993	114 min	Overcast with drizzle	0.81	64.30
Baily Head	Chinstrap Penguin Colony	62°58′01″S 60°30′21″W	M300	DJI H20T	Feb 5th, 2022	574	39 min	Overcast and Foggy	3.58	28.25
			M300	DJI H20T	Feb 5th, 2022	145	8 min	Overcast and Foggy	3.87	14.46

BAE GdC: Spanish Antarctic Base “Gabriel de Castilla”; M300: DJI Matrice 300; M2EA: Mavic 2 Enterprise Advanced; GSD: ground sampling distance.

**Table 2:** Information on UAV surveys, study locations, and flight conditions at study locations on Livingston Island.

Location	Key aspects	Central Coordinates	UAV	Sensor	Date	# Images	Flight Time	Weather Conditions	Average GSD (cm/px)	Covered Area (ha)
Hannah Point	Chinstrap and Gentoo Penguin Colony	62°39′16″S 60°36′48″W	M300	DJI L1	Feb 11th, 2022	120	4 min	Overcast with drizzle	2.15	11.65
			M300	DJI L1	Feb 25th, 2022	207	13 min	Partly cloudy	2.54	14.32
Johnsons Dock	Glacial Dome with Ice-lobes	62°39′37″S 60°22′03″W	M300	DJI H20T	Feb 14th, 2022	612	38 min	Overcast	7.60	124.27
			M300	DJI H20T	Feb 16th, 2022	597	21 min	Overcast	3.17	24.00
			M300	DJI L1	Feb 16th, 2022	117	9 min	Overcast	2.18	17.06
			M300	DJI H20T	Feb 16th, 2022	674	43 min	Overcast	6.29	92.24
Sally Rocks	Small Cluster of Rocks in the Water	62°42′09″S 60°25′46″W	M300	DJI H20T	Feb 15th, 2022	377	23 min	Overcast with drizzle	6.19	68.84
			M300	DJI L1	Feb 15th, 2022	309	24 min	Overcast with drizzle	3.26	54.37
Argentinian Cove	Glacial Dome with Ice-lobes. Snow Algae in some flights.	62°40′11″S 60°24′21″W	M300	DJI L1	Feb 14th, 2022	223	14 min	Partly cloudy	3.77	33.82
			M300	DJI H20T	Feb 14th, 2022	262	16 min	Partly cloudy	6.43	49.97
			M300	DJI H20T	March 5th, 2022	58	2 min	Overcast	1.32	1.06
BAE JCI	Spanish Antarctic Base	62°39′36″S 60°23′24″W	M300	DJI Zenmuse P1	Feb 12th, 2022	54	4 min	Overcast	2.42	13.41
Hurd Peninsula	Western Coast	62°40′33″S 60°21′59″W	VTOL	Sony A6000	Feb 28th, 2022	1004	56 min	Partly cloudy	7.26	1171.99
Charrúa Ridge	Glacial Dome with Ice-lobes	62°39′23″S 60°20′54″W	M300	DJI H20T	March 17th, 2022	641	40 min	Partly cloudy	7.44	162.33
Miers Bluff	Chinstrap Penguin Colony	62°43′12″S 60°26′11″W	M300	DJI H20T	March 2nd, 2022	835	31 min	Overcast	1.97	13.61
			M300	DJI L1	March 2nd, 2022	548	29 min	Overcast	2.71	24.86
			M300	DJI H20T	March 8th, 2022	232	9 min	Overcast	6.66	30.24
			M300	DJI L1	March 8th, 2022	307	18 min	Overcast	2.71	25.14

BAE JCI: Spanish Antarctic Base “Juan Carlos I”, M300: DJI Matrice 300; VTOL: vertical takeoff and landing UAV; M2EA: Mavic 2 Enterprise Advanced; GSD: ground sampling distance.

Hurd Peninsula is located along the southern coast of Livingston Island, and it can be divided into three primary geomorphological units: (i) the platform, a flat area where coastal cliffs separate this surface from the sea. Sally Rocks, a small cluster of rocks trending southwestward in South Bay^47^, and Miers Bluff formation, a 3 km thick succession of deformed turbiditic sedimentary rocks^49^, are notable examples within this geomorphological unit. (ii) The mountainous region in the southern portion of the peninsula, characterized by abrupt crests, steep slopes and summits; and (iii) the glacial dome located at the central part of the peninsula, covered by an ice-cap that at times flows radially towards the sea, forming various ice-lobes^50^. In this latter geomorphological unit, the surveyed areas include Johnsons Dock, Argentinian Cove, and the Charrúa Ridge.

Livingston Island harbors a highly diverse terrestrial and lacustrine flora and fauna^51^. Plant species are confined to ice-free areas, and are expanding in regions experiencing greater ice retreat, more favourable temperatures, and the influence of seabird colonies^51^. On Hurd Peninsula, Miers Bluff hosts a Chinstrap penguin colony on its coastal area. Hannah Point is a narrow peninsula situated on the southern coast of the island, characterized by a distinctive topography featuring a series of north-northwest cliffs that are separated from an open beach area by a steep slope^52^. It hosts a highly diverse fauna, including Chinstrap, Gentoo (*Pygoscelis papua*), and Macaroni (*Eudyptes chrysolophus*) penguin colonies, giant petrels (*Macronectes giganteus*), or Weddell seals (*Leptonychotes weddellii*), among others^53^. Furthermore, vegetation communities are of widespread interest as they comprise a variety of vascular plants, mosses, crustose lichens, and terrestrial algae^53,54^.

### UAV equipment and sensors

In this study, data collection was conducted using three different UAVs:

1. The DJI Matrice 300 RTK (M300) quadcopter, equipped with three different sensors: the DJI Zenmuse H20T, the DJI Zenmuse P1, and the DJI Zenmuse L1.
  - The DJI Zenmuse H20T sensor consists of a 20 MP optical RGB Zoom sensor with a 1/2.7” CMOS and a 12 MP optical RGB wide-angle sensor with a 1/2.3” CMOS. This sensor includes a detachable gimbal, allowing for a shutter speed of 1/8000 seconds. Based on previously established manufacturer laboratory conditions, this sensor has an accuracy of 0.2 m plus the distance to a vertical surface multiplied by 0.15%.
  - The DJI Zenmuse P1, integrated with a 45 MP full-frame sensor and with interchangeable lens (35 mm used in this case), features a 3-axis gimbal for intelligent oblique camera stabilization and a global mechanical shutter that allows for a shutter speed of 1/2000 seconds. Based on previously established manufacturer laboratory conditions, the Zenmuse P1 has a horizontal accuracy of 3 cm and a vertical accuracy of 5 cm, respectively.
  - The DJI Zenmuse L1 sensor integrates a high-precision IMU and a 20 MP CMOS sensor, that enables the capture of RGB optical images with a mechanical shutter speed of 1/2000 seconds and an electronic shutter speed of 1/8000 seconds, all stabilized with a 3-axis gimbal system. The accuracy settings of this sensor were measured under previously established manufacturer laboratory conditions, achieving 5 cm vertical and 10 cm horizontal for the optical RGB module, while achieving 0.025° (roll/pitch) and 0.15° (yaw) accuracy for the high-precision IMU.
2. The ATYGES FV1, a fixed-wing VTOL that is easily deployable due to its vertical landing and take-off capability. It was equipped with the Sony Alpha 6000, a 24.3 MP CMOS sensor, which enables automatic flight up to three continuous hours.
3. The DJI Mavic 2 Enterprise Advanced (M2EA), which included an additional RTK (real-time kinematic positioning) module for precise georeferencing. Equipped with a 48 MP, 1/2” CMOS optical RGB sensor, this quadcopter achieved a horizontal accuracy of 1 cm and a vertical accuracy of 1.5 cm under previously established manufacturer laboratory conditions.

### UAV data collection

The Spanish Civil Aviation regulations, which are overseen by the Spanish Agency for Aviation Safety (AESA), were adhered to throughout the entire operational procedure involving UAVs. Licensed UAV pilots followed the recommendations published by Hodgson and Koh^55^ and the Scientific Committee on Antarctic Research (SCAR)^56^ to ensure minimal disturbance to wildlife in areas where UAV operations posed minimal environmental risks. Flights were pre-programmed using UgCS desktop software (SPH engineering, Latvia, v.4.14) for both the M300 and the M2EA. This software accounted for the terrain’s topographic characteristics and set constant parameters for the flights conducted in this study, including flight height above sea level (ASL), speed, time, trajectory, and capture overlap (80% front and side overlapping). Flying at a constant altitude resulted in the continuous variation of the Ground Sample Distance (GSD), which produced the average GSD value for all individual captures in Tables 1, 2. The VTOL flights were prepared with the QGroundControl software (Dronecode Project, Inc. the Linux Foundation). As a general practice, Ground Control Points (GCPs) were not collected since most of the study locations were either difficult to access or large enough for full coverage, and the manufacturer’s RTK accuracy was considered to be of the highest precision. However, a short test flight with GCPs was conducted at the BAE GDC (Deception Island) to compare the photogrammetric process’s accuracy with and without GCPs (not included in the repository). In this test, six GCPs were evenly distributed around the Antarctic base and consisted of easily identifiable black-and-white rectangular targets visible from the air. To carry out post-processing kinematic (PPK) georeferencing using the DJI Zenmuse P1, a Reach RS2 + RTK GNSS antenna (EMLID) was employed as a reference station, supplying horizontal and vertical measurements of 4 mm + 1 ppm and 8 mm + 1 ppm, respectively. This reference station, with its precise coordinates manually entered at a known point, measures errors and transmits corrections to the sensor. Using PPK, it’s possible to establish base coordinates with centimeter-level accuracy, even without real-time corrections. Once the coordinates are obtained, they can be manually input as base coordinates. In addition, the antenna height must be manually entered, calculated as the distance between the marker and the bottom of the receiver plus 134 mm, representing the receiver’s height to the antenna reference point. When the antenna is securely placed over the marked point on the tripod, its position can be determined.

### Structure from Motion (SfM) photogrammetry

The software Pix4D Mapper (Pix4D SA, Lausanne, Switzerland, v.4.8.3) was used to generate optical RGB orthomosaics for each UAV flight. This software has frequently been employed for UAV-based terrestrial applications, with comprehensive methodological evaluations regarding the use of S*f*M photogrammetry for the generation of topographic products^57–59^.

Upon importing all UAV captures, a sparse point cloud was constructed during the “image alignment” step, employing a full image scale for keypoints (equivalent to half of the image size), pairing images using the “aerial grid or corridor” model (which uses triangulation for matching every two neighbouring images, with a maximum of five image pairs per Manual Tie Point (MTP)), and following an automatic standard camera calibration method, optimizing all prior internal and all external sensor (rotation and position) parameters. In addition, Geometrically Verified Matching was selected since it is useful when many similar features are present in the image, such as homogeneous surfaces corresponding to large coastal areas, vegetation, or snow coverages.

Subsequently, a “3D dense cloud” was generated using the aligned captures. The point cloud densification was carried out, considering half of the original image size, with an optimal point density and requiring a minimum of 3 points per match. Filtering of the point cloud was performed in Pix4D ray cloud, aiming to remove outliers in poorly resolved areas. The 3D textured mesh was created with a high-resolution setting, considering a maximum Octree depth of 14 and a decimation criterion of a maximum of 5000 triangles. An interpolated Digital Surface Model (DSM) was then derived from the “3D dense cloud”, with a spatial resolution equivalent to the image capture Ground Sampling Distance (GSD), and noise and surface smoothing filters were applied. Finally, the orthomosaic was rendered using the DSM as a reference surface. The coordinate system used for all photogrammetric process-derived products was WGS84/UTM zone 20 S (EPSG: 32720).

## Data Records

The ShetlandsUAVmetry dataset is publicly available at the figshare repository^60^. The data has been organized based on study locations, with two top-level folders named “Deception Island” and “Livingston Island”, respectively. Note that these two top-level folders are divided into multiple ZIP files to facilitate easy downloading directly from the repository^60^.

Within the main top-level folders, different subfolders named after the flight sites (Tables 1, 2) contain both the original RAW data and the photogrammetric products (including DSMs, Point Clouds, 3D mesh, and RGB orthomosaics) resulting from each UAV survey on the corresponding dates. Each DSM and RGB orthomosaic is stored in geotiff (.tif) format. On the other hand, each 3D mesh cloud is stored in FBX (.fbx) format and each point cloud is provided in LAS (.las) format. Each final product file has been named according to the following format: *YYYYMMDD_I_LOC_SEN_PRO_FN*; where *YYYYMMDD* indicates the flight date (*YYYY* for year, *MM* for month, and *DD* for day); *I* indicates the island where the UAV survey was performed (*D* for Deception Island and *L* for Livingston Island), while *LOC* denotes the specific study location (full name); *SEN* denotes the sensor or UAV platform used to collect the data (*L1*, *P1*, *H20T*, *A6000*, or *M2EA*); *PRO* indicates the photogrammetric product (*OR* for the orthomosaic, *DSM* for the Digital Surface Model, *PC* for the Point Cloud, *3D* for the 3D mesh, and *RP* for the accuracy report); and *FN* denotes the flight number in case there are more than one flight at each location. The original RAW data folder has been named as *RAW data – SEN*, where *SEN* corresponds to the sensor employed, as mentioned earlier.

**Fig. 2:**
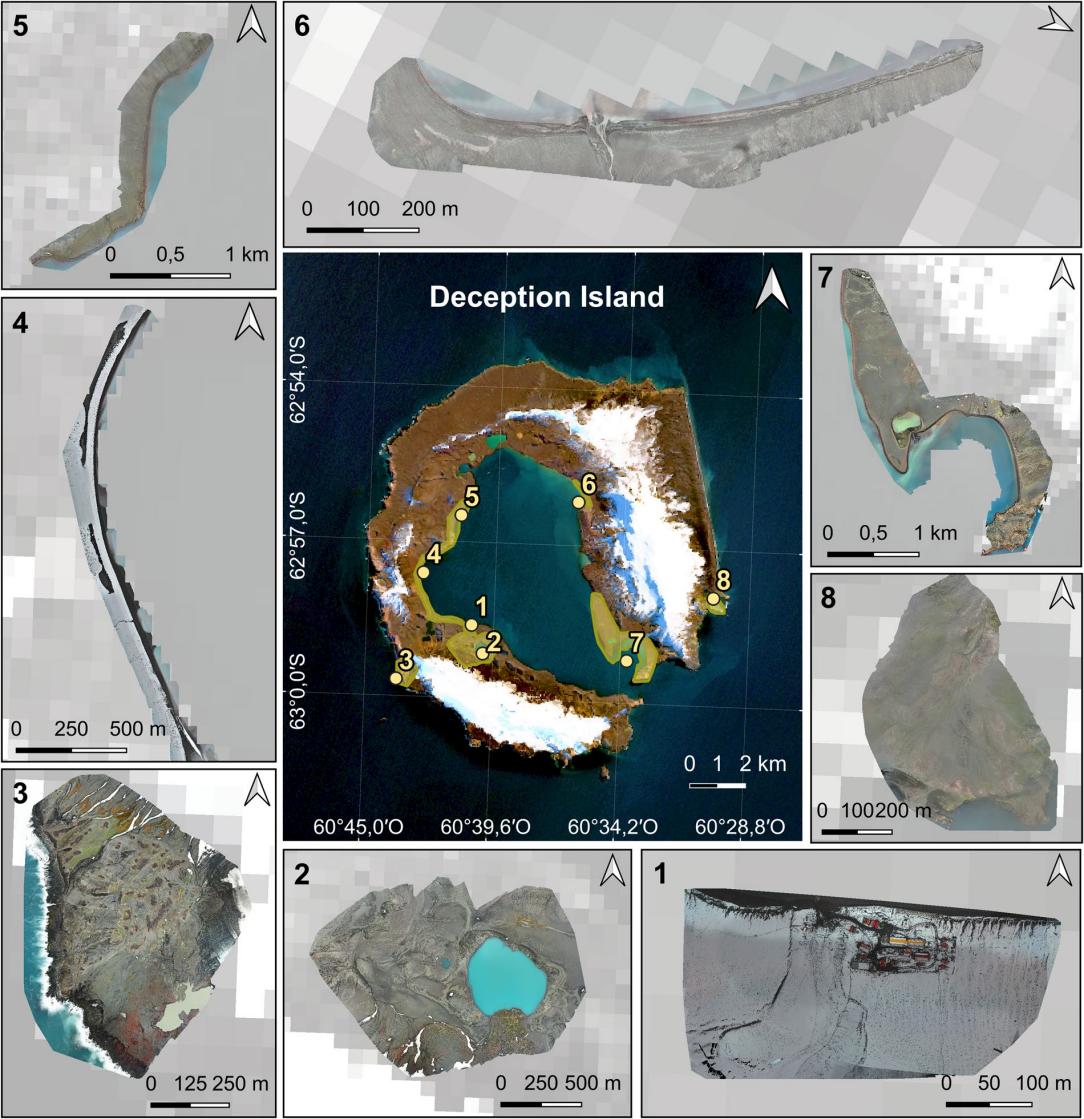
Overview of the optical RGB orthomosaics generated after the photogrammetric process for each study site at Deception Island (central panel, Sentinel 2 A scene of Deception Island on 17 March 2023). Marked in yellow, the footprints of the UAV surveys coverage. Numbers indicate locations: (1) BAE Gabriel de Castilla, (2) Crater Lake, (3) Vapour Col, (4) Fumarole Bay, (5) Murature Formation, (6) Pendulum Cove, (7) Whalers Bay, and (8) Baily Head.

**Fig. 3:**
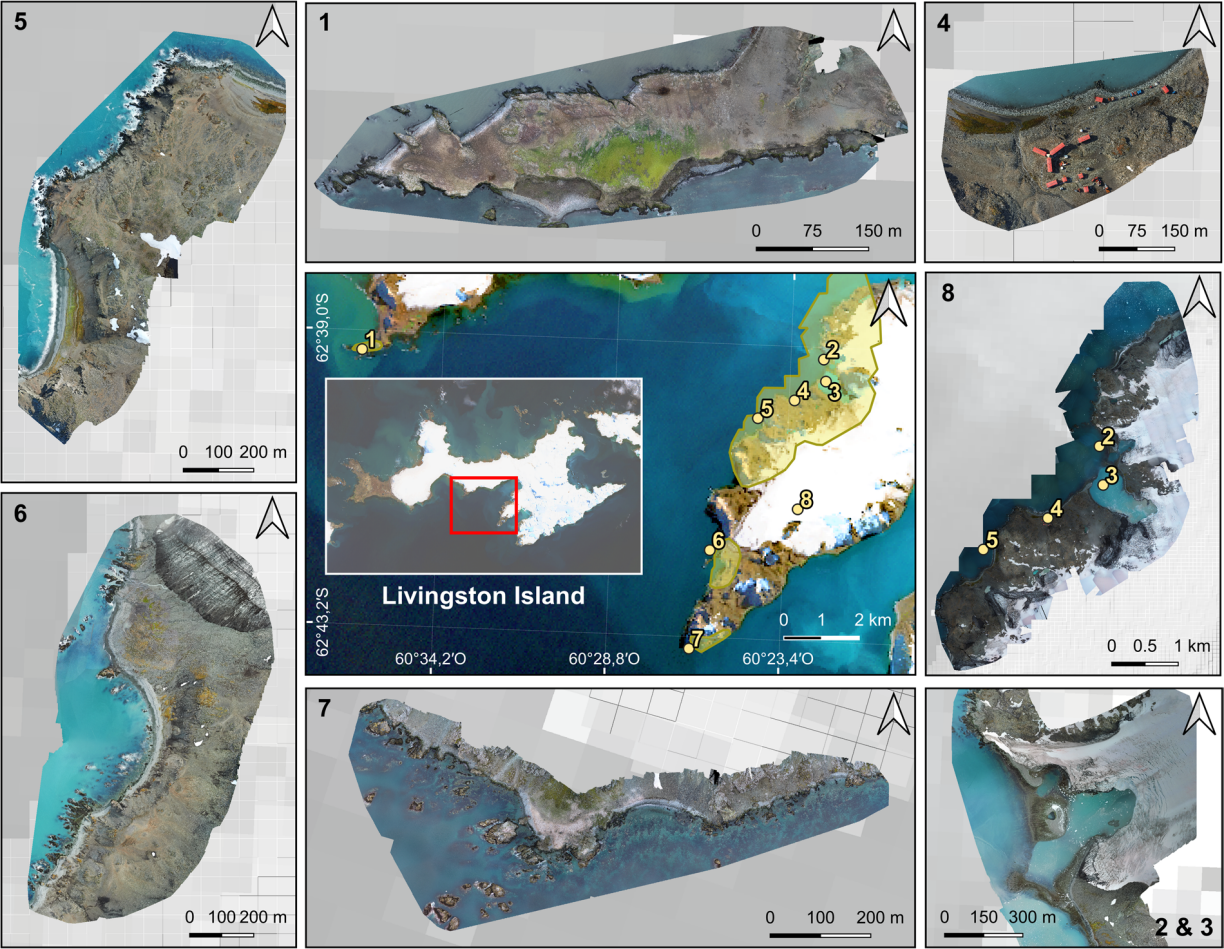
Overview of the optical RGB orthomosaics generated after the photogrammetric process for each study site at Livingston Island (central panel, Sentinel 2 A scene of Livingston Island on 17 March 2023). Marked in yellow, the footprints of the UAV surveys coverage. Numbers indicate locations: (1) Hannah Point, (2) Charrúa Ridge, (3) Johnsons Dock, (4) BAE Juan Carlos I, (5) Argentinian Cove, (6) Sally Rocks, (7) Miers Bluff, and (8) Hurd Peninsula.

Accompanying each UAV-processed photogrammetric product is a detailed accuracy report automatically generated when processing the data with Pix4D Mapper Software. The report includes the following: (i) a summary that reveals some characteristics of the final products; (ii) a preview of the photogrammetric results for the specific UAV flight; (iii) calibration details, which provide information about the initial image positions, computed tie points positions, overlapped areas between captures, and absolute uncertainties derived from the camera’s position and orientation; (iv) an accuracy assessment that includes the bundle block adjustment details and geolocation information; and finally, (v) a detailed description of the initial processing details (system information, coordinate systems, and processing options), point cloud densification details (processing options and results), as well as the DSM, orthomosaic, and index details (processing options). A comprehensive description of the accuracy assessment is provided in the “Technical Validation” Section. For Deception Island, a general overview of all covered areas is represented in Fig. 2, while for Livingston Island, it is shown in Fig. 3.

## Technical Validation

When working with an RTK module connected to the Reach RS2 + RTK GNSS antenna, it provides real-time corrections to enhance the accuracy of GPS/GNSS positioning during the UAV flight, although deploying GCPs on the ground is the only truly reliable way to assess the accuracy of UAV surveys. However, these real-time corrections enable obtaining more precise location data compared to conventional GPS systems. By using the RTK system, the positioning error is significantly reduced, thereby improving the accuracy of the photogrammetric results^61–63^.

The quality of photogrammetric products has been evaluated in terms of overlapping, reprojection, and geolocation accuracy, based on the information provided by the automatically generated accuracy report after the S*f*M photogrammetry process. On the one hand, the absolute camera position and orientation uncertainties provide an estimation of the error associated with the camera position and orientation parameters used for 3D reconstruction^64^. The lower the error, the higher the expected accuracy in the 3D reconstruction and georeferencing of mapped regions. On the other hand, the relative camera position and orientation uncertainties reflect the relative position and orientation of each camera in relation to the other cameras in the system, which is crucial for stereo triangulation and the generation of accurate 3D models^65^. Lower uncertainty values indicate higher precision in estimating the relative positions and orientations of the cameras, which in turn, can lead to more accurate 3D reconstruction.

Regarding the quality of overlapping, the report provides an indicative graph depicting the number of computed images per pixel in the final photogrammetric products. Results are considered photogrammetrically accurate when there are at least 5 images covering each pixel, as greater levels of overlapping can enhance accuracy, especially when working with finer image resolutions^66,67^. However, even in properly overlapped areas, problematic model outputs can occur when monitoring homogeneous surfaces^68,69^ or in adverse flight conditions (e.g. poor illumination or reduced visibility), since certain parts of the monitored area may be omitted. In addition, the Bundle Block Adjustment Details report section provides a detailed overview of the block adjustment process and the results obtained for each specific flight, allowing for the evaluation of the quality of the results. Multiple factors are considered, including the number of captures, the overlap between them, calibration points, and other inputs that help minimize differences between the sensor positions and orientations and the observed 3D points in the images. As accuracy statistics in this process, the report provides the mean reprojection error, reflecting the differences between the estimated and observed coordinates in different capture points of the UAV. Finally, the geolocation accuracy of the photogrammetric results is determined by analyzing the variance of absolute and relative geolocation. The variance of absolute geolocation indicates the uncertainty associated with the absolute geolocation of the results in relation to a global reference coordinate system. On the other hand, the variance of relative geolocation refers to the precision in estimating the spatial relationships between the mapped points. In both cases, lower values indicate higher precision in assigning geographic coordinates to the photogrammetric results.

Table 3 synthesizes the most relevant statistics for each UAV flight. After a thorough evaluation, it can be considered that all the photogrammetric products available in this repository are of exceptional precision. In all the photogrammetric projects processed, the mean reprojection error, that represents the distance between the initial position of each 3D point in the point cloud and its reprojection onto the 2D plane, is not bigger than 0.312 pixels except for the *20220224_L_BAEJCI_H20T* flight, which has an error of 0.466 pixels, although this error can still be considered as very accurate (generally, a mean reprojection error of less than 1 pixel is good). Regarding the employed sensors, the DJI Zenmuse P1 demonstrates to be better prepared for photogrammetric work, as reflected in lower geolocation RMSE values for each axis. It is followed by the DJI Zenmuse L1, which exhibits a very similar precision and is intended to complement the LiDAR sensor. The lowest statistics are found in flights carried out with the DJI M2EA, which is equipped with lower-performance sensors. In specific cases where unfavourable weather conditions were present or that included small sectors with no data in homogeneous snow water surfaces, the final products resulted in a smaller coverage than planned due to a reduced number of matches found between captures. These concerns are out of the scope of this repository, although in such cases, the analysis of derived products, such as point clouds or DSMs, may reflect the uncertainties inherent in the photogrammetric process^70^. The ShetlandsUAVmetry repository includes point clouds corresponding to each flight, which have undergone visual inspection to eliminate unexpected outliers, particularly in regions with more uniform characteristics, in order to guarantee the quality of the end products. The criteria outlined in Vieira *et al*.^69^ was followed, distinguishing between high-quality areas characterized by dense point clouds with no significant gaps, medium-quality areas where sporadic 3D errors may occur, and low-quality areas marked by patches where the point cloud resolution was poorly resolved. Finally, there is also the particular case of the *20220228_L_HURDPENINSULA_VTOL* flight, which covered a much larger area where, especially in water-covered areas, the photogrammetry process is less effective resulting in a deterioration of the final statistics.

**Table 3:** Summary of the main accuracy parameters of each UAV processed flight.

Product File	Camera Optimization (%)	Mean Reprojection Error (pixel)	Geolocation RMSE Error (m)		
			x	y	z
20220123_D_BAEGDC_P1	0.09	0.128	0.160975	0.186654	0.230183
20220124_D_CRATERLAKE_H20T	~0.00	0.272	0.976489	2.085300	2.329690
20220126_D_VAPOURCOL_H20T	0.02	0.312	1.179908	1.207040	1.704424
20220126_D_VAPOURCOL_L1	~0.00	0.146	0.208139	0.437946	0.484928
20220129_D_WHALERSBAY_H20T	0.02	0.260	1.330316	2.134870	3.604695
20220130_D_PENDULUMCOVE_M2EA	~0.00	0.164	0.865549	0.934217	3.756522
20220131_D_MURATURE_H20T	0.02	0.282	1.183381	1.671117	3.455522
20220201_D_FUMAROLEBAY_M2EA	0.03	0.175	0.900218	1.235584	2.818348
20220205_D_BAILYHEAD_H20T_1	0.04	0.248	1.386090	1.823700	1.352403
20220205_D_BAILYHEAD_H20T_2	~0.00	0.260	0.468765	1.957895	4.618313
20220211_L_HANNAHPOINT_L1	0.03	0.161	0.233058	0.390806	0.535136
20220225_L_HANNAHPOINT_L1	0.08	0.180	0.335242	0.476700	0.405695
20220214_L_JOHNSONSDOCK_H20T	0.02	0.274	2.184510	1.883845	3.794386
20220216_L_JOHNSONSDOCK_H20T_1	0.07	0.292	1.780134	2.543517	0.436305
20220216_L_JOHNSONSDOCK_L1	0.05	0.193	0.329352	0.301189	0.223212
20220216_L_JOHNSONSDOCK_H20T_2	0.03	0.282	2.454837	2.109086	2.445415
20220215_L_SALLYROCKS_H20T	0.15	0.307	1.1834464	2.642794	1.667040
20220215_L_SALLYROCKS_L1	0.08	0.182	0.197805	0.229927	0.506056
20220214_L_ARGENTINIAN_L1	0.74	0.185	0.217703	0.177878	0.898142
20220214_L_ARGENTINIAN_H20T	0.42	0.296	1.325917	2.407743	1.133520
20220305_L_ARGENTINIAN_H20T	0.51	0.362	1.624312	0.552698	0.062526
20220212_L_BAEJCI_P1	~0.00	0.124	0.082258	0.127282	0.107766
20220228_L_HURDPENINSULA_VTOL	0.06	0.173	2.006990	9.334304	1.791880
20220317_L_CHARRUARIDGE_H20T	0.23	0.281	2.965240	3.119623	1.443291
20220302_L_MIERSBLUFF_H20T	0.24	0.292	0.628972	1.984800	0.345665
20220302_L_MIERSBLUFF_L1	0.18	0.158	0.642075	0.414596	0.659426
20220308_L_MIERSBLUFF_H20T	0.08	0.300	3.734628	1.459845	1.744645
20220308_L_MIERSBLUFF_L1	0.17	0.182	0.261708	0.351944	0.487074

## Usage Notes

### Visual quality check

The raw data is available in the repository for further processing. However, as described in previous sections, data processing has been performed to provide the resulting photogrammetric products with the highest possible quality. Table 4 summarizes the authors’ assessment following the quality check conducted on the processed results.

**Table 4:** Quality check and assessment of the photogrammetric products available in the ShetlandsUAVmetry repository.

Product File	OR	DSM	3D	PC
20220123_D_BAEGDC_P1	HQ	HQ	HQ	HQ
20220124_D_CRATERLAKE_H20T	HQ	HQ	HQ	HQ
20220126_D_VAPOURCOL_H20T	HQ	HQ	HQ	HQ
20220126_D_VAPOURCOL_L1	MQ	MQ	MQ	Combination of HQ, MQ, and LQ areas.
20220129_D_WHALERSBAY_H20T	HQ	HQ	HQ	HQ
20220130_D_PENDULUMCOVE_M2EA	HQ	HQ	HQ	HQ
20220131_D_MURATURE_H20T	HQ	HQ	HQ	HQ
20220201_D_FUMAROLEBAY_M2EA	HQ	HQ	HQ	Combination of HQ and MQ areas.
20220205_D_BAILYHEAD_H20T_1	MQ with foggy	HQ	MQ	MQ
20220205_D_BAILYHEAD_H20T_2	MQ with foggy	HQ	MQ	MQ
20220211_L_HANNAHPOINT_L1	HQ with small XY shit	LQ	LQ	Combination of HQ, MQ, and LQ areas.
20220225_L_HANNAHPOINT_L1	HQ	HQ	HQ	Combination of HQ and MQ areas.
20220214_L_JOHNSONSDOCK_H20T	HQ	HQ	HQ	HQ
20220216_L_JOHNSONSDOCK_H20T_1	HQ	HQ	HQ	Combination of HQ and MQ areas
20220216_L_JOHNSONSDOCK_L1	HQ with small XY shit	LQ	LQ	LQ
20220216_L_JOHNSONSDOCK_H20T_2	HQ	HQ	MQ	Combination of HQ and MQ areas
20220215_L_SALLYROCKS_H20T	HQ	HQ	HQ	HQ
20220215_L_SALLYROCKS_L1	HQ	HQ	HQ	Combination of HQ and MQ areas
20220214_L_ARGENTINIAN_L1	HQ with XY shit	HQ	HQ	Combination of HQ and MQ areas
20220214_L_ARGENTINIAN_H20T	HQ	HQ	HQ	HQ
20220305_L_ARGENTINIAN_H20T	HQ	HQ	HQ	Combination of HQ and MQ areas
20220212_L_BAEJCI_P1	HQ	HQ	HQ	HQ
20220228_L_HURDPENINSULA_VTOL	HQ	HQ	HQ	Combination of HQ and MQ areas
20220317_L_CHARRUARIDGE_H20T	HQ	HQ	HQ	HQ
20220302_L_MIERSBLUFF_H20T	HQ	HQ	HQ	HQ
20220302_L_MIERSBLUFF_L1	HQ	HQ	HQ	HQ
20220308_L_MIERSBLUFF_H20T	HQ	HQ	HQ	HQ
20220308_L_MIERSBLUFF_L1	HQ	HQ	HQ	HQ

Abbreviations: orthomosaic (OR), Digital Surface Model (DSM), 3D mesh (3D), Point Cloud (PC), High Quality (GQ), Medium Quality (MQ), and Low Quality (LQ).

### Data Visualization and post-processing

Orthomosaics and DSMs can be visualized in any GIS software, such as QGIS (QGIS Development Team, Geographic Information System, Open Source Geospatial Foundation Project, v.3.16.14, https://qgis.org), or SAGA GIS^71^ v.7.9.0 (https://saga-gis.sourceforge.io/en/index.html). To work with point clouds, it is recommended to use CloudCompare v.2.12.4 (http://www.cloudcompare.org/).

### Wildlife census

UAVs are increasingly being used to monitor wildlife in the main Antarctic colonies, particularly for the seabird census^23,72,73^. These colonies have undergone significant population changes in recent decades due to the impact of climate change. Specifically, artificial intelligence (AI) techniques are capable of automatically counting the number of individuals in each population. It is recommended to use the published and available code at https://github.com/obkorolev/penguin_iron_paper^22^ which has previously been tested for the chinstrap penguin census in the Vapour Col colony on Deception Island. The flights available in this repository that can be processed using this technique include those conducted in Vapour Col, Baily Head, Hannah Point, and Miers Bluff.

### Security

One of the major challenges and problems that UAV operators in Antarctica have to face when carrying out fieldwork is the lack of GPS map references in the area, especially for flight planning and execution. For this reason, the vast majority of flights are conducted blindly, which can lead to accidents like the one that occurred during the flight *20220228_L_HURDPENINSULA_VTOL*, that was scheduled for three hours but crashed before when it collided with a terrain elevation. The data published in this repository can serve as a basis for conducting flights safely, as some flight planning softwares such as UgCS use the DSM to consider the topography of the terrain.

## Data Availability

SfM photogrammetry was performed using Pix4D Mapper (Pix4D SA, Lausanne, Switzerland, v.4.8.3) software, following the instructions provided in the user manual, which can be found at https://support.pix4d.com/hc/en-us/sections/360003718992-Manual. The processing templates for each UAV sensor are included in the repository, in a dedicated top-level folder named “Pix4D templates”.
